# Classical β-Lactamase Inhibitors Potentiate the Activity of Daptomycin against Methicillin-Resistant Staphylococcus aureus and Colistin against Acinetobacter baumannii

**DOI:** 10.1128/AAC.01745-16

**Published:** 2017-01-24

**Authors:** George Sakoulas, Warren Rose, Andrew Berti, Joshua Olson, Jason Munguia, Poochit Nonejuie, Eleanna Sakoulas, Michael J. Rybak, Joseph Pogliano, Victor Nizet

**Affiliations:** a University of California—San Diego School of Medicine, La Jolla, California, USA; b Sharp Healthcare System, San Diego, California, USA; c University of Wisconsin—Madison School of Pharmacy, Madison, Wisconsin, USA; d Department of Biological Sciences, University of California—San Diego, La Jolla, California, USA; e Eugene Appelbaum College of Pharmacy, Wayne State University, Detroit, Michigan, USA; f Skaggs School of Pharmacy, University of California—San Diego, La Jolla, California, USA

**Keywords:** beta-lactamase inhibitor, cathelicidin, colistin, daptomycin, peptide antibiotic

## Abstract

We asked whether beta-lactamase inhibitors (BLIs) increased the activity of daptomycin (DAP) against methicillin-resistant Staphylococcus aureus (MRSA), the peptide antibiotic colistin (COL) against the emerging Gram-negative nosocomial pathogen Acinetobacter baumannii, and the human host defense peptide cathelicidin LL37 against either pathogen. DAP and LL37 kill curves were performed with or without BLIs against MRSA, vancomycin-intermediate S. aureus (VISA), and heterogeneous VISA (hVISA). COL and LL37 kill curves were performed against A. baumannii. Boron-dipyrromethene (BODIPY)-labeled DAP binding to MRSA grown with the BLI tazobactam (TAZ) was assessed microscopically. The combination of COL plus TAZ was studied in a murine model of A. baumannii pneumonia. TAZ alone lacked *in vitro* activity against MRSA or A. baumannii. The addition of TAZ to DAP resulted in a 2- to 5-log_10_ reduction in recoverable MRSA CFU at 24 h compared to the recoverable CFU with DAP alone. TAZ plus COL showed synergy by kill curves for 4 of 5 strains of A. baumannii tested. Growth with 20 mg/liter TAZ resulted in 2- to 2.5-fold increases in the intensity of BODIPY-DAP binding to MRSA and hVISA strains. TAZ significantly increased the killing of MRSA and A. baumannii by LL37 *in vitro*. TAZ increased the activity of COL in a murine model of A. baumannii pneumonia. Classical BLIs demonstrate synergy with peptide antibiotics. Since BLIs have scant antimicrobial activity on their own and are thus not expected to increase selective pressure toward antibiotic resistance, their use in combination with peptide antibiotics warrants further study.

## INTRODUCTION

Treatment of multidrug-resistant (MDR) bacterial infections is posing an increasing challenge for clinicians worldwide. The “ESCAPE” pathogens (vancomycin-resistant Enterococcus faecium [VRE], methicillin-resistant Staphylococcus aureus [MRSA], Clostridium difficile, Acinetobacter baumannii, Pseudomonas aeruginosa, and carbapenem-resistant Enterobacteriaceae) have required us to expand our antimicrobial therapeutic repertoire through either the development of new drugs or “thinking outside the box” to identify novel applications of our existing antibiotic options. In addition, the predictive value of traditional antibiotic susceptibility testing for patient outcomes remains limited and fails to fully replicate *in vivo* conditions (1). For example, the activity of the macrolide antibiotic azithromycin may be significantly underestimated using standard bacteriological media compared to media used for eukaryotic tissue culture cell growth, such as Roswell Park Memorial Institute 1640 (RPMI 1640) medium (2–4). Additional data show nonlethal but clinically significant effects of antistaphylococcal β-lactams on MRSA and ampicillin on vancomycin-resistant E. faecium, affording these drugs a useful role as adjunctive therapy in difficult infections (5–7).

We have found that carbapenems and penicillins are better than cephalosporins in potentiating the activity of the peptide antibiotic daptomycin (DAP) against MRSA (8). We noted similarities of the structures of these compounds to the structures of the classic β-lactamase inhibitors (BLIs) tazobactam (TAZ), sulbactam (SLB), and clavulanic acid. We hypothesized that the BLIs themselves may also exert similar effects in potentiating peptide antibiotics. We focused specifically on the Gram-positive therapeutic agent DAP against MRSA and the Gram-negative therapeutic agent colistin (COL) against A. baumannii. To extend our studies to potential innate immune potentiation, we examined the effects of BLIs on the activity of the endogenous antimicrobial peptide human cathelicidin LL37 against both MRSA and A. baumannii.

(This work was presented in part at the 55th Interscience Conference on Antimicrobial Agents and Chemotherapy [ICAAC], September 2015, San Diego, CA [24].)

## RESULTS

### The peptide antibiotic COL and TAZ are synergistic against A. baumannii.

**FIG 1:**
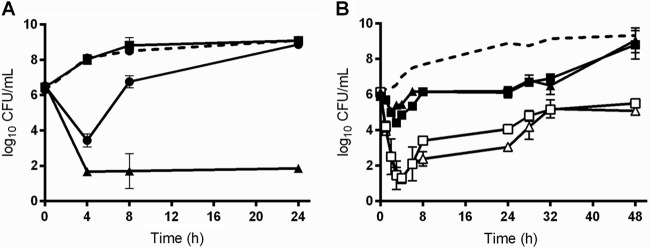
Time-kill curves demonstrating synergistic killing of COL plus TZB against Acinetobacter baumannii. (A) Kill curve of static sub-MICs against A. baumannii strain AB5075 (dashed line, growth control; black circles, COL 0.4 mg/liter; black squares, TAZ 20 mg/liter; black triangles, COL 0.4 plus TAZ 20 mg/liter). (B) Simulated *in vitro* PK/PD modeling against another clinical A. baumannii strain (dashed line, growth control; black squares, COL 2.5 mg/kg/day; black triangles, COL 5 mg/kg/day; white squares, COL 2.5 mg/kg/day plus TAZ 500 mg q8h; white triangles, COL 5 mg/kg/day plus TAZ 500 mg q8h).

Kill curve assays were performed against a total of 6 A. baumannii strains. Strain AB was examined by both time-kill assays (Fig. 1A) and pharmacokinetic/pharmacodynamic (PK/PD) modeling simulating COL at 2.5 or 5 mg/kg of body weight/day alone or combined with TAZ at 500 mg every 8 h (q8h) (Fig. 1B). COL alone resulted in ∼3-log_10_ growth from the starting inoculum, but the combination showed rapid killing early, with regrowth at later time points. AB5057 and 4 additional clinical strains were examined by standard kill curves, and the results are shown in Fig. S1 in the supplemental material. Synergy was noted where each agent alone resulted in no killing but the two-drug combination resulted in bactericidal activity against AB5075 and three of the four clinical strains tested. Interestingly, the presence of TAZ at 20 mg/liter in the medium reduced the COL MIC by only 1 dilution for 2 of the 4 strains tested (including AB5075) and did not change the MIC for 2 strains.

### BLIs enhance the activity of the peptide antibiotic DAP against MRSA.

**FIG 2:**
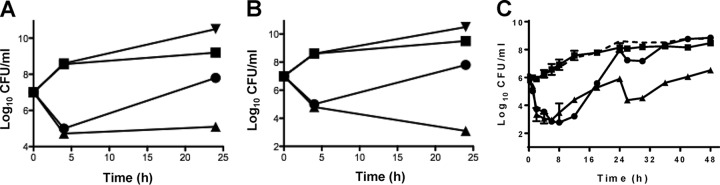
Time-kill curve demonstrating synergistic killing of DAP plus beta-lactamase inhibitors. (A) Kill curve of static sub-MICs of DAP plus TAZ against MRSA strain Sanger 252 (black down triangles, growth control; black circles, DAP 2 mg/liter; black squares, TAZ 20 mg/liter; black up triangles, DAP 2 mg/liter plus TAZ 20 mg/liter); (B) Kill curve of static sub-MIC of DAP plus SLB against MRSA strain Sanger 252 (black down triangles, growth control; black circles, DAP 2 mg/liter; black squares, SLB 20 mg/liter; black up triangles, DAP 2 mg/liter plus SLB 20 mg/liter); (C) Simulated *in vitro* PK/PD modeling of DAP plus TAZ against daptomycin-nonsusceptible VISA D712 (dashed line, growth control; black circles, DAP 6 mg/kg/day; black squares, TAZ 500 mg q8h; black up triangles, DAP 6 mg/kg/day plus TAZ 500 mg q8h).

Antimicrobial susceptibility results against S. aureus bacteria are shown in Table 1. Kill curve assays performed by using subinhibitory antibiotic concentrations alone and in combination showed synergy of the BLI TAZ (Fig. 2A) or SLB (Fig. 2B). Vancomycin-intermediate S. aureus (VISA) strain D712 was examined by PK/PD modeling simulating DAP at 6 mg/kg/day and TAZ at 500 mg every 8 h (comparable to the TAZ exposure with piperacillin-TAZ at 4.5 g every 8 h). As anticipated, TAZ or DAP monotherapy against this VISA strain displayed minimal activity, with bacterial counts at 24 and 48 h being similar to those of the growth control (∼2-log_10_ growth above that of the starting inoculum). The combination of TAZ plus DAP resulted in bacterial counts that were 2 log_10_ units lower than those with either monotherapy, although this was approximately the starting inoculum at 24 and 48 h (Fig. 2C).

**TABLE 1:** Bacterial strains used in this study

Strain	Strain description	Antimicrobial susceptibility (MIC [mg/liter])^*a*^				
		VAN	DAP	COL	PB	TAZ
AB5075	A. baumannii	NA	NA	1	1.5	64
AB1	A. baumannii	NA	NA	0.75	1	>64
AB2	A. baumannii	NA	NA	1	0.5	>64
AB3	A. baumannii	NA	NA	1.5	1.5	>64
AB4	A. baumannii	NA	NA	1	1	64
D712	VISA	4	4	NA	NA	>32
D592	hVISA	2	1	NA	NA	>32
MRSA Sanger	MRSA	1	0.5	NA	NA	>32

a NA, not applicable; PB, polymyxin B.

### BLIs enhance DAP binding to MRSA and hVISA bacterial cell membranes.

**FIG 3:**
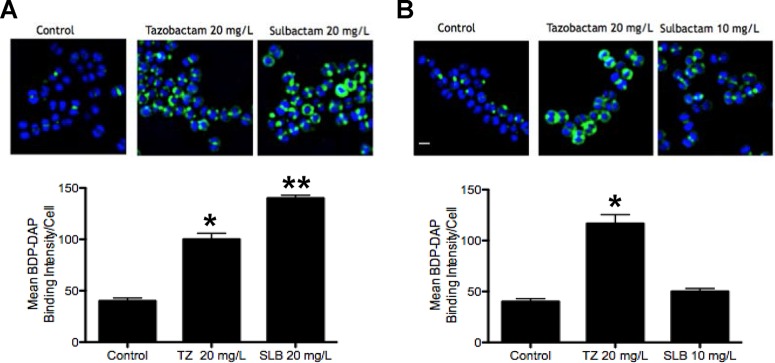
Increased binding of BODIPY-labeled daptomycin (BDP-DAP) by tazobactam (TZ) or SLB for MRSA Sanger 252 (A) or hVISA D592 (B). Cells were incubated for 20 min with BODIPY-DAP at 8 mg/liter (Sanger 252) or 16 mg/liter (D592) after growth to an OD_600_ of 0.5 in antibiotic-free medium (control) or with TAZ or SLB. Error bars show standard errors of the means for 3 analyzed fields. *, *P* < 0.001 (A) or *P* < 0.05 (B); **, *P* < 0.0001 (A).

Sample microscopic fields and graphic quantitation of boron-dipyrromethene (BODIPY)-DAP binding are shown for MRSA Sanger 252 (Fig. 3A) and heterogeneous VISA (hVISA) strain D592 (Fig. 3B). The concentrations of TAZ and SLB of 20 mg/liter were chosen to be at or close to the maximum concentration of drug in serum (*C*_max_) when these compounds are administered clinically with piperacillin and ampicillin, respectively. At this concentration, MRSA Sanger showed enhanced binding to BODIPY-DAP. However, for hVISA D592, the conditions were kept the same for TAZ, but the concentration of SLB was reduced to 10 mg/liter. The lower SLB concentration resulted in BODIPY-DAP binding close to that for the antibiotic-free control.

### BLIs enhance the activity of the human cathelicidin LL37.

**FIG 4:**
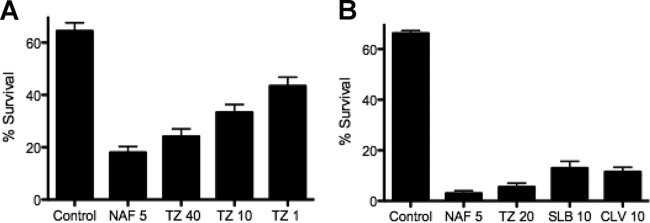
(A) LL37 killing assays displaying 2-h survival of VISA strain D712 (LL37 at 128 μM) after growth overnight in antibiotic-free medium (control) or in medium containing nafcillin (NAF) or tazobactam (TZ) at the indicated concentrations (milligrams per liter). (B) Similar experiment with hVISA D592 utilizing LL37 at 64 μM and nafcillin, tazobactam, SLB, or clavulanic acid (CLV).

We have previously shown that the growth of MRSA with concentrations of β-lactam antibiotics at a fraction of the MIC enhances the vulnerability of MRSA to killing by host cationic defense peptides, including the human cathelicidin LL37 (5, 6). VISA strain D712 was grown with subinhibitory nafcillin concentrations or various concentrations of TAZ (1, 10, and 40 mg/liter), spanning a concentration range achieved *in vivo* with the currently approved dosing of piperacillin-TAZ, and then treated with LL37 (128 μM) for 2 h, with percent survival being shown in Fig. 4A. With nafcillin serving as a previously characterized positive control, increasing concentrations of TAZ stimulated increased bacterial killing by LL37. A similar study was performed for hVISA D592, utilizing LL37 (64 μM) after growth with TAZ, SLB, or clavulanic acid, with a similar enhancement of killing (Fig. 4B).

**FIG 5:**
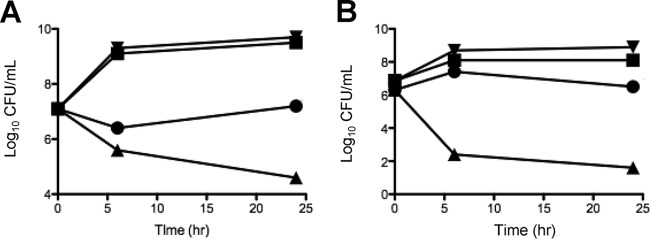
Time-kill curve demonstrating synergistic killing with host defense cationic peptide LL37 plus beta-lactamase inhibitors. (A) MRSA Sanger 252 (black down triangles, growth control; black circles, LL37 64 uM; black squares, TAZ 20 mg/liter; black up triangles, LL37 64 mM plus TAZ 20 mg/liter); B) Acinetobacter baumannii AB5075 (black down triangles, growth control; black circles, LL37 2 mM; black squares, TAZ 20 mg/liter; black up triangles, LL37 2 mM plus TAZ 20 mg/liter.

To complement these studies, MRSA Sanger 252 and A. baumannii AB5075 were studied with conventional kill curves to examine LL37 and TAZ synergy (Fig. 5A and B, respectively), using subinhibitory concentrations of each drug alone or in combination. TAZ exposure alone did not change bacterial growth compared to that with the medium control, whereas LL37 exposure alone resulted in stasis against MRSA and a 2-log_10_ kill with regrowth to the starting inoculum for A. baumannii. However, the combination of both TAZ and LL37 at one-quarter their respective MICs resulted in a synergistic ∼2.5-log_10_ kill for MRSA (Fig. 5A) and a rapid and sustained 4-log_10_ kill for A. baumannii (Fig. 5B).

### BLIs do not prevent the emergence of DAP resistance in MRSA using *in vitro* passage.

It was previously demonstrated that β-lactams slow the emergence of DAP nonsusceptibility in MRSA by preventing *mprF* mutation selection (9). MRSA was passaged serially with DAP alone as well as with TAZ, SLB, or oxacillin as a positive control. Table 2 demonstrates that unlike oxacillin, the BLIs were unable to prevent an increase in the DAP MIC over 28 days.

**TABLE 2:** Effect of combination passage with daptomycin *in vitro* on selection of daptomycin-nonsusceptible MRSA

Drug exposure	Day 28 DAP MIC (mg/liter)					
	Replicate 1	Replicate 2	Replicate 3	Replicate 5	Replicate 5	Median
DAP	8	12	24	32	32	24
DAP + tazobactam	1	16	16	16	32	16
DAP + sulbactam	8	32	32	32	64	32
DAP + oxacillin	0.02	0.05	1	3	3	1

### TAZ enhances COL activity in a murine model of A. baumannii pneumonia.

**FIG 6:**
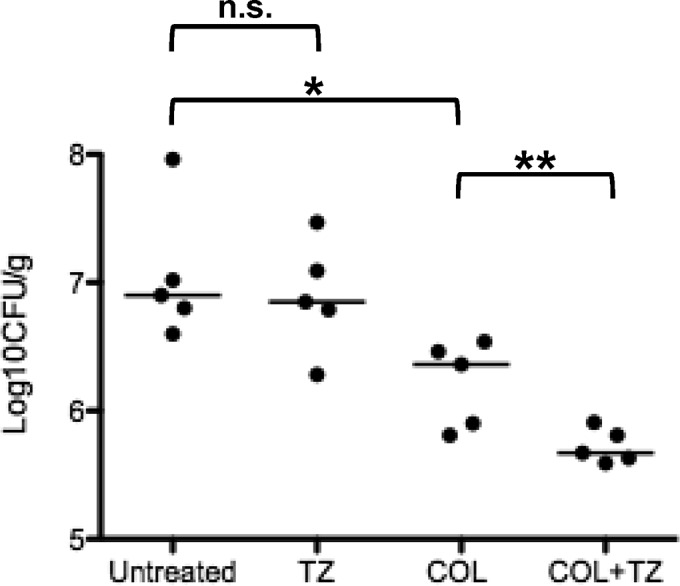
Murine model of A. baumannii pneumonia in BALB/c mice (5 per group) treated for 24 h with COL, tazobactam (TZ), both COL and tazobactam, or saline and enumeration of bacteria in harvested lungs (CFU per gram). Horizontal bars represent the medians for each group. *, *P* = 0.008 versus the control; **, *P* = 0.047 versus COL; n.s., not significant.

The combination of COL plus TAZ was tested in a murine model of A. baumannii (AB5075) pneumonia. An enhancement in bacterial clearance from lung tissue was observed upon the addition of TAZ compared to that with COL alone (Fig. 6).

## DISCUSSION

Expanding our knowledge of the use of our currently available antibiotics, especially against drug-resistant Gram-negative bacteria, is more important than ever before as the number of antibiotic-resistant bacterial infections increases worldwide (10). Among the Gram-negative bacteria, A. baumannii is particularly problematic since none of the newly developed antibiotics focused on expanding treatment options for infections by Gram-negative pathogens provide any further benefit above what had already been available. Ceftolozane-TAZ provides enhanced activity against Pseudomonas aeruginosa and common extended-spectrum β-lactamases (11), whereas another new combination, ceftazidime-avibactam, employs a novel non-β-lactam BLI to counteract the carbapenemases that are becoming more common in Enterobacteriaceae (12). For MRSA, we have been fortunate to have several new drugs added to the clinical therapeutic repertoire in the past decade to build upon the waning efficacy of vancomycin, but treatment of the most severe infections remains problematic, often requiring combination antimicrobial therapy (5, 6, 13, 14).

BLIs with β-lactam structures were previously characterized to have some antibacterial activity on their own, including activity against Neisseria spp., Acinetobacter spp., Burkholderia, and even Borrelia burgdorferi. Early studies by Urban et al. characterized the activities of SLB and TAZ against A. baumannii and determined that at lower concentrations, the effect was due to β-lactamase protection, but at higher concentrations, the effect was due to the binding of penicillin binding protein 2 (PBP2) > PBP1, with consequential effects on cell wall synthesis (15). However, a more recent study showed that the activity of SLB against A. baumannii was much more complex (16). Those researchers found that SLB bound PBP1 > PBP3 > PBP2 but that the total effect of SLB activity was “greater than the sum of the parts” that could be assayed with conventional PBP studies. Those authors hypothesized that binding to lower-molecular-weight PBPs or other factors involved in cell wall synthesis may play a role in the collective activity of SLB against A. baumannii (16).

The goal of this study was to examine whether the β-lactam molecular structure of traditional BLIs that affect cell wall synthesis through cell wall binding would enhance peptide antibiotic activity (COL against A. baumannii and DAP against MRSA). We found a potentiation of COL activity by TAZ against multiple strains of A. baumannii *in vitro*. The concentrations of TAZ chosen in kill curve assays are representative of serum concentrations obtained in patients with conventional approved dosing of piperacillin-TAZ, while COL concentrations were approximately one-half the MIC. The synergy observed in kill curve assays was corroborated by using PK/PD simulated modeling methods as well. TAZ enhanced the killing activity of A. baumannii by human cathelicidin LL37 by using both traditional kill curve experiments and pretreatment sensitization methods. Finally, TAZ improved the activity of COL in a murine model of A. baumannii pneumonia employing the virulent multidrug-resistant strain AB5075. Of interest, growth of COL-dependent A. baumannii was completely abolished in the presence of easily achievable concentrations of TAZ, suggesting a possible role of BLIs in reducing the emergence of COL dependence or as a combination agent with COL in serious A. baumannii infections.

DAP activity against MRSA, hVISA, and VISA was enhanced by BLIs, as evidenced by data from kill curve studies, PK/PD modeling, and the demonstration of enhanced DAP membrane binding by microscopy. Similarly to findings with A. baumannii, the activity of the host defense peptide cathelicidin LL37 was enhanced by BLIs. These studies underscore a poorly appreciated phenomenon in which β-lactam molecules may exert useful pharmacodynamic effects against β-lactam-resistant organisms, which may be exploited in combination antimicrobial therapy to provide more enhanced antibacterial potency and/or to reduce the emergence of antimicrobial resistance.

While the precise mechanisms of how BLIs enhance DAP and LL37 activities against MRSA, VISA, and hVISA are unknown, these compounds likely influence cell wall synthesis and metabolism through PBP binding rather than protection of the primary β-lactam through β-lactamase inhibition. This is supported by our observations of cell wall thickness using electron microscopy (our unpublished observations). A recent study by Smith et al. also supports this notion by showing that ceftolozane-TAZ enhances the activity of DAP against the DAP-susceptible MRSA isolate used in their study (17). Ceftolozane by itself shows little to no activity against MRSA and does not enhance the activity of DAP. The addition of TAZ enhances DAP activity, suggesting that TAZ and ceftolozane may exhibit complementary PBP binding against MRSA that allows enhanced DAP activity.

These findings hold some important implications. First, they add further support to an already growing line of data demonstrating a very profound and global “seesaw effect” between β-lactam and peptide antibiotics, which spans across multiple species, both Gram positive and Gram negative. It appears that the β-lactam structure, and, consequentially, PBP binding and inhibition of cell wall synthesis, extends the seesaw effect to BLIs. Our preliminary evaluation of non-β-lactam BLIs shows that the seesaw effect does not extend to this class of compounds (G. Sakoulas, unpublished observations).

Second, these data support the clinical availability of BLIs as solo agents and not solely as compounds to be coadministered with β-lactam antibiotics. The availability of BLIs alone would allow them to be coadministered with peptide antibiotics and reduce collateral damage in selecting resistant bacteria, which has been documented with the broad use of β-lactams and BLIs in combination. Third, these data further underscore the importance of combination antibiotic therapy against the most resistant bacterial infections.

Limitations of this study include the fact that it involved the evaluation of a small number of strains, and not all experiments were carried out with all the available classical BLIs. An *in vivo* study was not performed to assess the effect of DAP plus BLI because DAP activity in animal models is excellent and therefore difficult to build upon, unlike the modest activity of COL. Most importantly, this phenomenon awaits clinical study, although dissecting the true clinical effects of BLIs alone will be limited, as these drugs are not available for use without a concomitant beta-lactam. While some evidence supports these data by showing a clinical benefit of adding SLB to COL for the treatment of A. baumannii pneumonia (18), a more thorough clinical evaluation is needed.

In summary, BLIs appear to enhance the activities of both exogenous and endogenous peptide antibiotics against MRSA and A. baumannii. While BLI activities are more modest than those of ceftaroline and antistaphylococcal beta-lactams in potentiating DAP activity and preventing DAP resistance against MRSA, they represent a novel starting point for designing future therapeutic antibiotic treatments against drug-resistant Gram-positive and Gram-negative pathogens.

## MATERIALS AND METHODS

### Bacterial strains and *in vitro* susceptibility and synergy tests.

Bacterial strains used in this study are described in detail in Table 1, including the well-characterized virulent strain A. baumannii AB5075 (19). Susceptibility testing was performed by using standard broth microdilution methods with cation-adjusted Mueller-Hinton II broth (MHB). Kill curves were performed with MHB in triplicate by utilizing the specified concentrations of DAP versus MRSA or COL versus A. baumannii, with or without the classical BLI TAZ or SLB (2, 13). The concentrations of COL (2) and DAP (13) utilized were based on previously reported data. TAZ and SLB concentrations were chosen to be just below the maximal serum concentration with standard dosing for the respective beta-lactam–BLI combination drug dosing (20, 21). Aliquots were obtained at 0, 3 to 6, and 24 h and serially diluted, and 10 μl was plated onto LB plates. CFU were counted after 24 h, and the number of CFU per milliliter was calculated. At 24 h, aliquots of combination antibiotics were spun down and concentrated 10-fold in fresh LB broth to bring the limit of detection to 10 CFU/ml (1.0 log_10_ CFU/ml). In kill curves, synergy was defined as the achievement of a >2-log_10_ reduction in CFU compared to that of the most active single agent (22). A. baumannii COL susceptibility testing was also performed with MHB containing TAZ at 20 mg/liter.

### Effects of BLIs on DAP bacterial membrane binding.

The ability of DAP to bind to membranes of MRSA Sanger 252 and hVISA D592 was determined by using BODIPY-labeled DAP (Cubist Pharmaceuticals, Lexington, MA) as previously described (5, 6). Briefly, bacteria were grown to an optical density at 600 nm (OD_600_) of 0.5 in antibiotic-free LB broth or in LB broth containing 10 to 20 mg/liter TAZ or SLB. Cultures were subsequently incubated for 20 min with 16 mg/liter BODIPY-DAP. Microscopy and quantification were performed by using methods described previously (5, 6).

### Effects of BLIs on cathelicidin LL37 killing of MRSA and A. baumannii.

Bacteria were grown overnight in antibiotic-free LB broth or in LB broth containing TAZ or SLB (10 to 20 mg/liter). Bacteria were spun down, washed once in sterile phosphate-buffered saline (PBS), and subjected to 2-h LL37 killing assays at the specified concentrations in RPMI medium supplemented with 10% LB broth, as previously described (5). Results were determined in quadruplicate and expressed as the percentage of surviving bacteria compared to the number of bacteria in the starting inoculum. Standard synergy assays for LL37 were also performed with RPMI supplemented with 10% LB broth, with sampling at 0, 4, and 24 h in triplicate. Results were expressed at log_10_ CFU per milliliter versus time.

### *In vitro* PK/PD modeling.

VISA strain D712 was analyzed by *in vitro* PK/PD modeling simulating DAP at 6 mg/kg daily and TAZ at 500 mg every 8 h alone or in combination by using previously reported methods for simulating the clearance of antibiotics with two different half-lives (23). A. baumannii 2166 was analyzed for killing by COL at 2.5 mg/kg/day and 5 mg/kg/day alone or in combination with TAZ at 500 mg every 8 h.

### Serial passage *in vitro* to examine preventing a loss of DAP susceptibility in MRSA by BLIs.

Isolated colonies of hVISA strain D592 were passaged for 28 days with escalating concentrations of DAP, as outlined previously (9), in medium containing no antibiotic or a consistent concentration of SLB, TAZ, or oxacillin (positive control). In this fashion, the concentration of DAP was increased stepwise, while the concentration of the BLI or oxacillin was held constant, as previously described (9). Secondary antibiotics were added at the following concentrations according to the average free-drug concentration in human serum [*fC*_avg_ *=* (*fC*_max_ *+ fC*_min_)/2]: 16 mg/liter for oxacillin, 12 mg/liter for TAZ, and 21 mg/liter for SLB. Five distinct, independent, replicate passages were assessed for each antibiotic combination tested. DAP MICs were evaluated weekly via broth microdilution using MHB supplemented with 50 mg/liter Ca^2+^.

### A. baumannii murine pneumonia model.

A murine model of A. baumannii AB5075 pneumonia and COL treatment was performed as previously described (2). A. baumannii AB5075 was grown in LB broth overnight and resuspended in sterile PBS to an OD_600_ of 0.5 (∼5 × 10^8^ CFU/ml), and 30 μl was administered intratracheally to BALB/c mice (25 g). One hour later, therapy was begun with either 0.1 ml PBS × 1 dose, TAZ at 20 mg/kg in 0.1 ml PBS q8h × 3 doses, COL at 8 mg/kg in 0.1 ml PBS × 1 dose, or both COL and TAZ administered intraperitoneally, with 5 animals/group. The animals were sacrificed at 24 h, and lungs were harvested, weighed, and homogenized in sterile PBS. Homogenates were plated in serial dilutions on Todd-Hewitt agar (THA) plates, and colonies were enumerated after 24 h to calculate the CFU per gram of tissue.

Animals were maintained in accordance with the American Association for Accreditation of Laboratory Animal Care Criteria, and the above-described studies were approved by the Animal Care and Use Committee (IACUC) of the University of California, San Diego.

## Supplementary Material

Supplemental material
