# Supplementary material for: Classical β-Lactamase Inhibitors Potentiate the Activity of Daptomycin against Methicillin-Resistant Staphylococcus aureus and Colistin against Acinetobacter baumannii
Source: Antimicrob Agents Chemother. 2017 Jan 24;61(2):e01745-16. doi: 10.1128/AAC.01745-16 (PMC5278754; doi:10.1128/AAC.01745-16)
Supplement: Supplemental material [file supp_61_2_e01745-16__index.html]

Classical β-Lactamase Inhibitors Potentiate the Activity of Daptomycin against Methicillin-Resistant Staphylococcus aureus and Colistin against Acinetobacter baumannii — Supplemental material 

# Classical β-Lactamase Inhibitors Potentiate the Activity of Daptomycin against Methicillin-Resistant Staphylococcus aureus and Colistin against Acinetobacter baumannii

## Supplemental material

- Supplemental file 1 -

  Fig. S1

  PDF, 101K
