# Supplementary material for: Classical β-Lactamase Inhibitors Potentiate the Activity of Daptomycin against Methicillin-Resistant Staphylococcus aureus and Colistin against Acinetobacter baumannii
Source: Antimicrob Agents Chemother. 2017 Jan 24;61(2):e01745-16. doi: 10.1128/AAC.01745-16 (PMC5278754; doi:10.1128/AAC.01745-16)
Supplement: Supplemental material [file AAC.01745-16_zac002175888s1.pdf]

Supplemental Figure S1

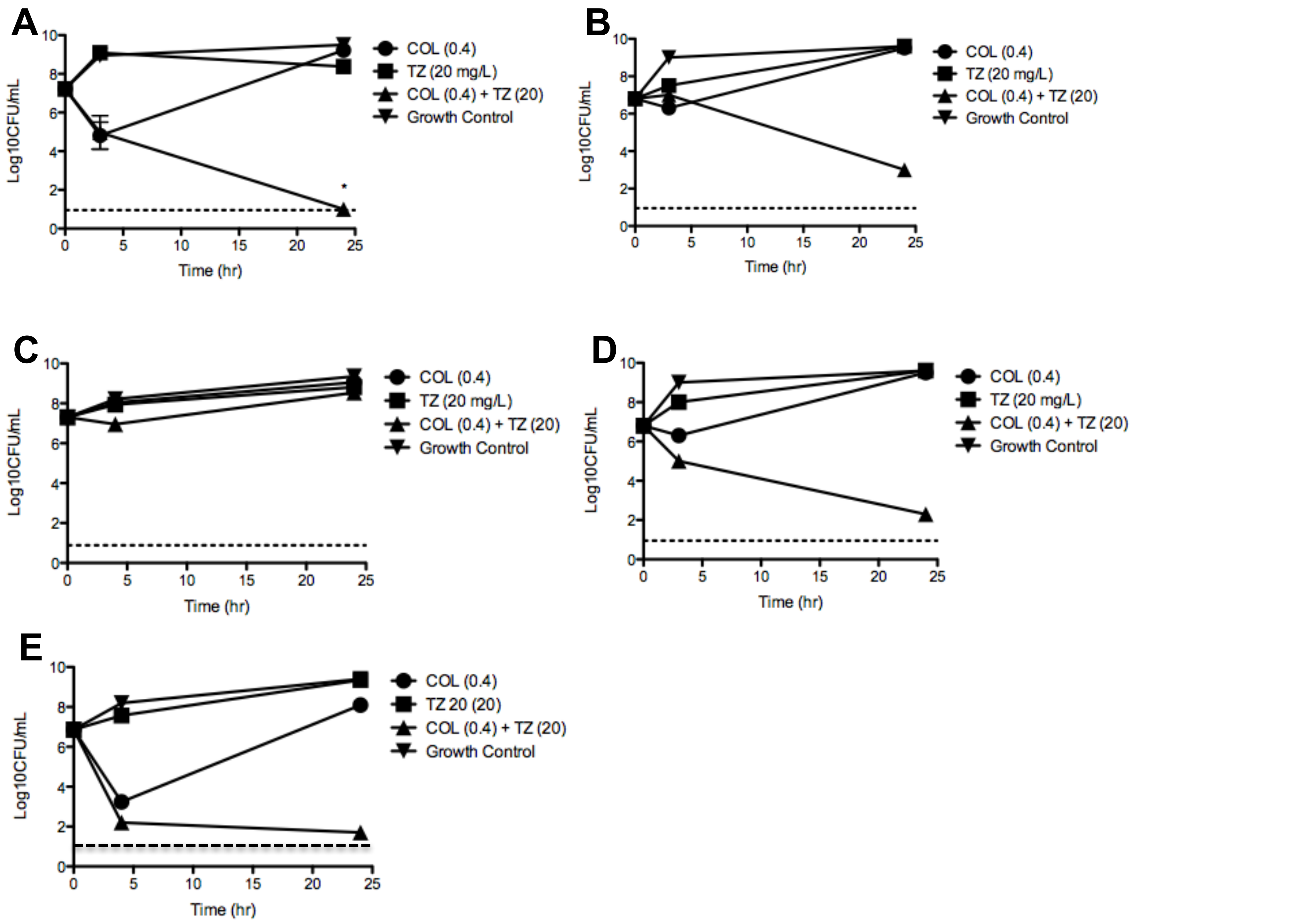

Supplemental Figure 1. Time kill curve demonstrating time-kill curves utilizing sub-MIC concentrations of colistin (COL, 0.4 mg/L) plus tazobactam (TZ, 20 mg/L) against 4 clinical *A. baumannii* strains (AB1-AB4, Panels A-D, respectively), and AB5075 (panel E). Four of the 5 strains showed synergy, whereas one strain (AB3) did not.
